# First Line Therapy in Mantle Cell Lymphoma—The Role of BTKi in the Initial Treatment of Transplant‐Eligible and ‐Ineligible Patients

**DOI:** 10.1002/hon.70073

**Published:** 2025-06-15

**Authors:** E. Silkenstedt, M. Dreyling

**Affiliations:** ^1^ Department of Medicine III LMU Hospital Munich Germany

**Keywords:** BTKi, first line, MCL

## Abstract

The development and implementation of new targeted and immunotherapeutic approaches in the treatment landscape of mantle cell lymphoma have already improved therapeutic options especially for refractory or relapsed disease. Regarding first‐line therapy, treatment strategies including novel agents are under investigation. In transplant‐eligible patients, a combined ibrutinib‐containing immunochemotherapy induction followed by ASCT was shown to be superior compared to the chemotherapy regimen +/− ASCT. Thus, we consider the addition of ibrutinib to first‐line therapy in younger MCL patients a new standard of care. For the group of older MCL patients ineligible for high‐dose therapy and ASCT, several targeted therapy approaches have been investigated in different studies in combination with immunochemotherapies or as single agents +/− other targeted therapies with promising results. This review summarizes the current standard of care for first‐line treatment of mantle cell lymphoma, highlighting the implementation of targeted treatment strategies, especially BTKi, in initial treatment strategies.

**Trial Registration:** ClinicalTrials.gov identifier: NCT06482684

## 1 Introduction

Mantle cell lymphoma (MCL) is clinically characterized by its heterogenous behavior with courses ranging from indolent cases that do not require therapy for years to highly aggressive MCL with very limited prognosis [1]. Patients typically present with lymphadenopathy of several sites, most of the patients are diagnosed with advanced stage disease (Ann Arbor stage III, IV). Extranodal manifestations occur in 90% of patients, including infiltration of bone marrow (53%–82%), blood (50%), liver (25%) and the gastrointestinal tract (20%–60%) [1, 2]. The spleen is enlarged in 40% of patients [1]. In some cases, leukemic manifestation in combination with massive splenomegaly is clinically prominent. These non‐nodal, leukemic cases are often characterized by a more indolent clinical course. Accordingly, in the WHO update of lymphoid malignancies, MCL was subdivided in two distinct categories [3]. Nodal MCL (80%–90% of cases) is characterized by unmutated immunoglobulin heavy chain variable region genes (IGHV), Sex‐Determining Region Y‐Box 11 (SOX11) overexpression and a generally more aggressive clinical behavior. Non‐nodal leukemic MCL (10%–20% of cases) typically displays mutated IGHV, SOX11 negativity and presents with indolent biological behavior. Histologically, besides “classical” MCL, pleomorphic and blastoid variants can be distinguished [3]. MCL with blastoid morphology often features high proliferation rates, displaying a more aggressive clinical course [3, 4].

Traditionally, MCL was associated with a poor prognosis with a median overall survival of 3–5 years. However, major advances in the treatment of MCL patients have been achieved over the last years.

In young and fit patients (≤ 65 years), a dose‐intensified concept containing an immunochemotherapy induction followed by a high‐dose consolidation regimen and ASCT constituted, until recently, the current standard of care [1, 5]. In a large randomized Phase 3 trial of the European Mantle Cell Lymphoma Network (MCL Younger), the administration of the R‐CHOP/DHAP regimen compared to administration of R‐CHOP alone prior to myelo‐ablative consolidation with ASCT more than doubled time to treatment failure (TTF) (109 vs. 47 months) [5]. A large randomized trial proved that consolidation by myeloablative radiochemotherapy followed by ASCT in first remission significantly prolonged PFS (3.3 vs. 1.5 years) and OS [6]. The value of high‐dose immunochemotherapy followed by ASCT was also demonstrated in the 15‐year update of the Nordic MCL2 study, reporting a median OS and PFS of 12.7 and 8.5 years, respectively in patients < 66 years treated with alternating courses of R‐maxi‐CHOP (dose‐intensified rituximab, cyclophosphamide, doxorubicin, vincristine, and prednisone) and high‐dose cytarabine (HIDAC), followed by ASCT consolidation [7].

Moreover, introduction of rituximab as maintenance therapy, especially for those patients not eligible for high‐dose therapy, significantly improved survival rates in this group of patients [8].

Achievement of prolonged duration of initial response is of great importance, taking into account that remission duration as induced by further treatment lines is generally shorter. This phenomenon was also observed in a pooled analysis (*n* = 370) in patients with relapsed MCL treated with the BTK inhibitor ibrutinib evaluating the depth and durability of response depending on the number of prior lines: Superior ORR (1 prior line 77.8% vs. > 1 prior line 66.8%), CR rate (1 prior line 36.4% vs. > 1 prior line 22.9%), median PFS (1 prior line 25.4 months vs. > 1 prior line 10.3 months) and median OS (1 prior line not reached vs. > 1 prior line 22.5 months) was observed when patients were treated with ibrutinib at first relapse compared to treatment at later relapse [9]. Ultimately, a long‐lasting first remission should be the primary goal in treatment of MCL and an intensified induction treatment followed by ASCT has proven to effectively achieve this. However, ASCT is also hampered by high acute and delayed toxicity. Thus, alternative first‐line treatment strategies without ASCT but including novel agents are under investigation.

This review summarizes the current treatment recommendations for first‐line treatment of mantle cell lymphoma, discussing the remaining value of ASCT and highlighting the implementation of targeted treatment strategies, especially BTKi, in initial treatment strategies.

## 2 Treatment

**FIGURE 1:**
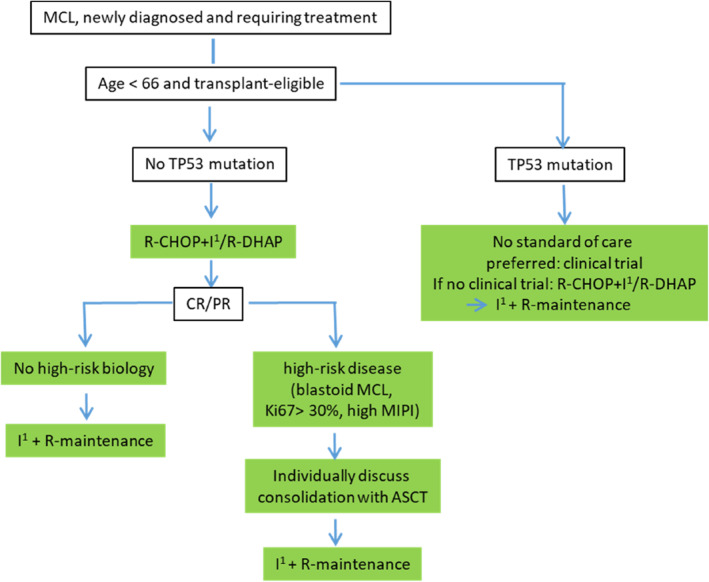
Suggested therapeutic algorithm for patients < 66 years. ^1^ = off‐label use; ASCT = autologous stem cell transplantation; CHOP = cyclophosphamid, doxorubicin, vincristine, prednisone; CR = complete remission; DHAP = dexamethasone, high‐dose cytarabine, cisplatin; I = ibrutinib; PR = partial remission; R = rituximab.

The clinical course of MCL is characterized by generally high initial response rates; however, early relapses are frequent and most patients follow an aggressive clinical course. Nevertheless, 10%–15% of patients present with a more indolent subtype. These cases are commonly characterized by a leukemic, non‐nodal lymphoma manifestation or a very low Ki67‐Index (< 10%). In these cases, watchful waiting under close monitoring is considered an appropriate strategy [10]. Yet, the majority of cases require an early treatment initiation even though advanced stage disease (stage III/IV) is still considered incurable. Figure 1 suggests a risk‐adapted treatment strategy for this group of patients.

### 2.1 Therapy in Patients ≤ 65 years

As shown in numerous studies, in young and fit patients (≤ 65 years), a dose‐intensified concept containing an immunochemotherapy induction followed by a high‐dose consolidation regimen and ASCT proved to achieve a high number of long‐lasting remissions and was, until recently, considered the standard of care [1, 5, 6, 7]. Rituximab maintenance after ASCT is currently considered standard of care for younger patients with MCL based on the results of a large Phase III trial showing a significantly improved PFS (83% vs. 64% after 4 years) and OS (89% vs. 80% after 4 years) after 3 years of rituximab maintenance compared to observation only [11].

However, it is important to note that this long‐term efficacy of ASCT in MCL was established in the pre‐rituximab era. The relative importance of ASCT versus no consolidation has not been prospectively evaluated in a randomized trial including modern induction therapy containing both Ara‐C and rituximab. Thus, a critical question to be addressed is whether the PFS and OS benefit of ASCT can nowadays be confirmed considering the availability of better salvage therapies, targeted treatments and optimized maintenance protocols. In a real‐world analysis, retrospective data from 4.216 patients with MCL in the Flatiron Health electronic record‐derived deidentified database diagnosed between 2011 and 2021, mostly in US community oncology settings, treatment outcomes and roles of ASCT and maintenance with rituximab in patients with previously untreated MCL were evaluated [12]. There was no significant association between ASCT and real‐world time to next treatment (TTNT) (HR 0.84; 95% CI, 0.68–1.03; *p* = 0.10) or OS (HR 0.86; 95% CI, 0.63–1.18; *p* = 0.4) among ASCT‐eligible patients (*n* = 1265). In contrast, the addition of rituximab maintenance to immunochemotherapy (rituximab‐bendamustine) resulted in a longer real‐world TTNT (HR 1.96; 95% CI, 1.61–2.38; *p* < 0.001) and OS (HR, 1.51; 95% CI, 1.19–1.92; *p* < 0.001) [12]. These findings may hint that long‐term FFS and OS benefit in the first‐line setting might be more likely be impacted by improved maintenance therapy rather than ASCT.

Importantly, ASCT is also hampered by high acute and delayed toxicity. This is particularly important when considering that the median age at diagnosis for MCL is 67; therefore, many MCL patients are at increased risk for toxicity after ASCT. Recently, the phase III TRIANGLE trial [13] evaluated the remaining value of ASCT for first‐line therapy: 870 patients < 65 years were treated with either the current standard of care including ASCT (arm A), the additional application of ibrutinib (arm A + I) or an ibrutinib‐combination without ASCT (arm I). After a median follow‐up of 53 months, FFS‐superiority of A + I over A was confirmed with 3‐year FFS 86% (A + I) versus 75% (A; one‐sided *p* = 0.0034, hazard ratio: 0.64). Similarly, the retrospectively calculated *p*‐value was also consistent with FFS‐superiority of I over A (*p* = 0.0102). In contrast, A + I failed to show FFS‐superiority over I in the total group (3‐year FFS A + I: 86% vs. I: 85%; one‐sided *p* = 0.28, hazard ratio: 0.87). FFS‐superiority of A over I was again not confirmed with 3‐year FFS 75% (A) versus 85% (I; one‐sided *p* = 0.9942, hazard ratio: 1.38), but subset analysis in the biological high risk patients suggested a benefit for the addition of ASCT. Finally, OS was prolonged in arms A + I and I with 3‐year OS of 90% in A + I (*p* = 0.0069, hazard ratio 0.61), and 91% in I (*p* = 0.0041, hazard ratio 0.59) compared with arm A (3‐year OS 85%) [14].

The TRIANGLE trial allowed rituximab maintenance added to all three trial arms according to national guidelines. As confirmed in an exploratory analysis, the addition of rituximab to maintenance therapy improved DOR in Arm I (HR 0.35). However, observed toxicity rates, particularly infections, were higher when combining maintenance therapy, especially when patients received additional high‐dose therapy, with 30% (Arm A + I) and 25% (Arm I) of patients developing at least one grade 3–5 infectious complication, compared to 13% (Arm A + I) and 12% (Arm I) of patients with ibrutinib maintenance only. In contrast, hematological toxicity (grade 3 or greater) was increased only in arm A (28% in the R maintenance group vs. 11% in the no R maintenance group), whereas comparable frequencies were observed in the other study arms (arm A + I: 46% in the R + I maintenance group vs. 52% in the I maintenance only group; arm I: 27% in the R + I group vs. 23% in the I only group) [15]. Importantly, the ibrutinib maintenance in the TRIANGLE trial was applied for a fixed duration of 2 years and the majority of patients were still in remission after completion of maintenance. Thus, re‐exposition to a BTKi in the relapse setting may be a valuable option. However, only limited data on a BTKi‐re‐challenge after a time‐limited ibrutinib treatment are currently available in MCL. It is important to note that it is possible that outcomes after relapse may be affected by previous BTKi exposure during induction and maintenance with lower responses to salvage BTKi.

In summary, the TRIANGLE trial demonstrated increased efficacy when adding ibrutinib to the previous standard approach with ASCT consolidation and shows non‐superiority of the standard treatment over an ibrutinib‐containing induction regimen without ASCT. As such, it defines a new standard of care in frontline treatment of young and fit MCL patients. Whether ASCT, with additional toxicity, still adds benefit to ibrutinib‐based treatment in a subset of patients, is not yet determined.

Based on growing evidence of the strong prognostic potential of MRD status predicting improved subsequent PFS for MRD‐negative patients at the end of induction and before high‐dose consolidation [5], the randomized Phase 3 trial of the ECOG‐ACRIN Cancer Research Group (ECOG‐ACRIN 4151; NCT03267433) compared ASCT followed by maintenance rituximab with maintenance rituximab alone (without ASCT) in MCL patients in minimal residual disease (MRD)‐negative first CR, irrespective of induction regimen. A first interim analysis showed that MCL patients in first CR with undetectable MRD at 1 in 10^−6^ sensitivity (uMRD6) did not benefit from consolidative auto‐HCT [16]. Patients who remained MRD‐positive after induction may benefit from PBSCT. Longer follow‐up will be important to confirm these findings.

MCL is a very heterogenous disease and high‐risk disease characteristics have been defined as high p53 expression and Ki‐67 > 30%, together with blastoid morphology, shown to be associated with a significantly shorter FFS and OS [17]. Despite optimal immunochemotherapy, high‐dose cytarabine and ASCT, younger patients with MCL with deletions or mutations of TP53 have an unfavorable prognosis, as reported in the European MCL Younger Trial [18, 19] and confirmed in the Nordic MCL2 and MCL3 trials [20]. Accordingly, the new lymphoma classification systems now recommend to determine Ki67 proliferation index and *TP53* status obligatory at first diagnosis [21, 22]. These patients especially benefit from addition of ibrutinib standard to first‐line treatment.

Another promising approach for this high‐risk group of patients was evaluated in a Phase 2 study of zanubrutinib, obinutuzumab, and venetoclax (BOVen) in untreated patients with MCL with a TP53 mutation [23]. This study recently reported an overall response rate of 96% (24/25) and a CR rate of 88%. After a median follow‐up of 28.2 months, the 2‐year progression‐free, disease‐specific, and overall survival were 72%, 91%, and 76%, respectively [23], indicating a very interesting treatment regimen for TP53 mutated patients, requiring ongoing evaluation.

It is an open question whether earlier application of immunotherapeutic approaches such as CAR‐T cells or T‐cell engagers may at least partially overcome the negative prognostic impact of TP53 alterations in these patients. This question is being addressed in the currently recruiting CARMAN trial, a randomized, controlled, international, multicenter, open‐label phase II trial evaluating the efficacy and safety of brexucabtagene autoleucel following an abbreviated induction (3 cycles of ibrutinib + rituximab (IR) and 2 cycles of Ibrutinib + R‐CHOP in patients not achieving at least a PR to IR), and 6 months ibrutinib maintenance starting 3 months post CAR‐T‐treatment (Arm A) as compared to standard of care chemoimmunotherapy combined with ibrutinib induction (patients ≤ 65 years will receive 3 cycles R‐CHOP + ibrutinib/3 cycles R‐DHAP alternating, followed by ASCT if appropriate and ibrutinib/rituximab maintenance [24]).

### 2.2 Therapy in Patients > 65 years

**FIGURE 2:**
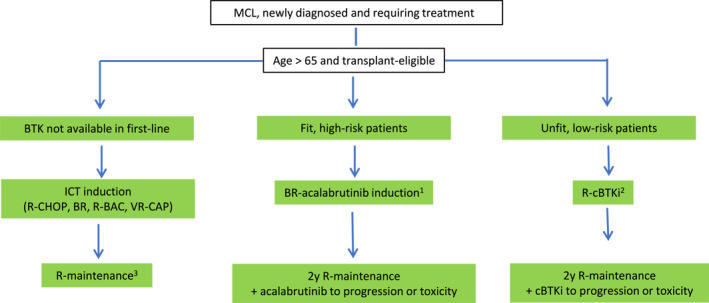
Suggested therapeutic algorithm for patients > 65 years. ^1^Registration expected end of 2025. ^2^Evidence from randomized trials currently with ibrutinib‐rituximab; zanubrutinib and acalabrutinib may represent reasonable alternatives. Not currently FDA or EMA approved. ^3^R‐maintenance not required post‐R‐BAC. B = bendamustine; BAC = bendamustine, low‐dose cytarabine; cBTKi = covalent Brutons Tyrosine Kinase inhibitor; CHOP = cyclophosphamid, doxorubicin, vincristine, prednisone; R = rituximab; VR‐CAP = bortezomib, rituximab, cyclophosphamide, doxorubicine, prednisone.

The group of the > 65‐year‐old patients ineligible for transplantation presents very heterogenous regarding physical and cognitive performance. A suggested therapeutic algorithm is depicted in Figure 2. Fit patients > 65 years should receive conventional immunochemotherapy followed by rituximab maintenance [8]. A combination of bortezomib, rituximab, cyclophosphamide, doxorubicine und prednisone (VR‐CAP) recently proved to be superior over R‐CHOP in a large international Phase III trial with a doubled overall survival (OS) after 82 months (90.7 vs. 45.7 months). However, hematologic toxicity (especially > grade 3 thrombopenia) was significantly increased in the experimental arm (57% vs. 6%), therefore a modification of schedule (bortezomib only day 1 and 4) is recommended [25]. The combination of rituximab, bendamustine and cytarabine (R‐BAC) offers another useful option. Yet, this regimen was accompanied by severe hematotoxicities and should therefore only be administered to very fit older patients with high‐risk features (e.g., blastoid variant, high LDH count) [26]. Alternatively, for patients not qualifying for such intensive therapy regimens, R‐bendamustine offers an appropriate alternative. This combination resulted in similar response rates (93% vs. 91%) compared to R‐CHOP and was numerically superior in progression‐free survival (PFS) (35 vs. 21 months) with a more favorable toxicity profile observed [27]. However, from our experience in clinical practice, BR is often associated with serious delayed infectious complications. In a UK real‐world data analysis, Grade 3–5 SAEs related to bendamustine were reported in 21.7% of patients receiving BR, with ∼50% due to infections, most commonly of respiratory and urinary tract origin and not related to neutropenia. Thirteen percent of patients stopped treatment because of bendamustine‐related toxicity, most commonly infection [28].

A large, randomized, European phase III trial compared rituximab maintenance to interferon maintenance, confirming superiority of rituximab as maintenance therapy. In this study, after 4 years, 58% of the patients receiving rituximab after induction therapy with R‐CHOP were in remission, compared to 29% in the interferon arm (*p* = 0.01). PFS and OS were also significantly improved in the rituximab arm (5‐year PFS R vs. IFN 51% vs. 22%, 5‐year‐OS R vs. IFN 79% vs. 59%) [29]. Similarly rituximab maintenance also resulted in superior PFS and OS rates in a large US‐based retrospective survey [11]. Based on these results, rituximab maintenance is now generally recommended.

Taken together, VR‐CAP and BR or R‐CHOP represent the current standard approaches in older patients not eligible for high‐dose therapy, who represent the majority of MCL patients. VR‐CAP should be, in our opinion, preferably chosen for patients with a higher risk‐profile such as high Ki67 expression or blastoid morphology. BR may be preferable especially in patients with a more indolent CLL‐like presentation.

The SHINE trial compared rituximab‐bendamustine (BR) plus ibrutinib versus BR alone in patients ≥ 65 years old and showed that the ibrutinib arm achieved a significantly prolonged median PFS (80.6 vs. 52.9 months) but similar OS (57% vs. 55% at 7 years) as compared to BR alone [30]. Acalabrutinib in combination with BR was evaluated in the Phase 3 ECHO trial, leading to a significant PFS benefit, including those patients with high‐risk features [31], and a trend toward improved OS especially after censoring COVID events [32].

The randomized Phase 2/3 ENRICH trial recently compared first‐line rituximab‐ibrutinib (IR) with R‐chemotherapy in patients ≥ 60 years and showed superiority of IR versus R‐chemo regarding PFS (65.3 vs. 42.4 months), especially when compared to R‐CHOP. Hematological toxicity was reduced with IR and quality of life was improved [33].

In summary, BR‐Acalabrutinib may represent a useful option particularly in fit high‐risk patients who can tolerate this triplet. Rituximab‐cBTKi without chemotherapy is deliverable in older patients with comorbidities.

## 3 Conclusion

Overall, cBTKi are increasingly integrated into first‐line algorithms and clinical routine. With the recently published results of the TRIANGLE trial, showing superiority of an ibrutinib‐containing regimens compared to the chemotherapy followed by ASCT, we consider the addition of ibrutinib to first‐line CHOP‐like therapy and rituximab maintenance in younger MCL patients a new standard of care in younger patients. In future, BR‐Acalabrutinib may represent useful options particularly in older but fit/robust patients who can tolerate this triplet. Rituximab‐cBTKi is an attractive option in older patients with comorbidities. However, the availability of cBTKi varies across Europe due to regulatory and reimbursement issues. If ibrutinib is unavailable, second generation cBTKi represent a reasonable replacement despite missing phase III data with zanubrutinib. Especially patients with TP53 aberrations benefit from the BTK‐based regimens; however, the contribution of intensified chemotherapy is debatable in this high‐risk subgroup and hampered by increased toxicity. Therefore these patients should, whenever possible, be treated within clinical trials.

## Conflicts of Interest

The authors declare no conflicts of interest.

## Peer Review

The peer review history for this article is available at https://www.webofscience.com/api/gateway/wos/peer-review/10.1002/hon.70073.

## Data Availability

The authors have nothing to report.
